# Author Correction: Emerging opportunities and challenges for the future of reservoir computing

**DOI:** 10.1038/s41467-024-54397-6

**Published:** 2024-11-18

**Authors:** Min Yan, Can Huang, Peter Bienstman, Peter Tino, Wei Lin, Jie Sun

**Affiliations:** 1 Theory Lab, Central Research Institute, 2012 Labs, Huawei Technologies Co. Ltd., Hong Kong SAR, China; 2 Photonics Research Group, Department of Information Technology, Ghent University, Gent, Belgium; 3 School of Computer Science, The University of Birmingham, Birmingham, B15 2TT United Kingdom; 4 Research Institute of Intelligent Complex Systems, Fudan University, Shanghai, 200433 China; 5 School of Mathematical Sciences, SCMS, SCAM, and CCSB, Fudan University, Shanghai, 200433 China

**Keywords:** Applied mathematics, Statistical physics, thermodynamics and nonlinear dynamics

Correction to: *Nature Communications* 10.1038/s41467-024-45187-1, published online 06 March 2024

The original version of this Article contained errors in the reference numbering in labels of Box 1, Fig. 1 and Fig. 3.

In Box 1, reference numbers [160], [161], [117], [118] have been replaced with [128], [129], [110], [111], correspondingly.

In Fig. 1, reference numbers [101], [102], [103], [90], [95], [133], [93], [97], [125], [127], [150] have been replaced with [94], [95], [104], [82], [88], [145], [85], [90], [137], [139], [161], correspondingly.

In Fig. 3, reference numbers [85], [90], [91], [92], [96], [97], [98], [99], [100], [101], [102], [103], [104], [105], [111] have been replaced with [86], [82], [83], [84], [89], [90], [91], [92], [93], [94], [95], [104], [105], [106], [98], correspondingly.

The last sentence of the second paragraph in the section ‘Application benchmarks or RC’ contained an error in one of the reference numbers. The correct version is: ‘The state-of-the-art of RC currently can reach a word error rate (WER) of 0.4% from memristor chip RC[35], and 0.2% from electronic RC[28].’

Box 1 contained an error where the word ‘between’ was misspelled and part of the caption for panel (b) in the figure within was omitted. These have been corrected.

These corrections have been implemented in both the PDF and HTML versions of the article.

